# Declining tuberculosis prevalence in Saharia, a particularly vulnerable tribal community in Central India: evidences for action

**DOI:** 10.1186/s12879-019-3815-8

**Published:** 2019-02-20

**Authors:** V. G. Rao, J. Bhat, R. Yadav, R. K. Sharma, M. Muniyandi

**Affiliations:** 1 ICMR -National Institute of Research in Tribal Health, (Indian Council of Medical Research), Nagpur Road, P.O. Garha, Jabalpur, 482 003 India; 2 ICMR -National Institute for Research in Tuberculosis, (Indian Council of Medical Research), No. 1, Mayor Sathiyamoorthy Road, Chetpet, Chennai, 600031 India

**Keywords:** Tuberculosis, Prevalence, Tribal, Saharia, PVTG, Madhya Pradesh, India

## Abstract

**Background:**

In spite of an alarmingly high tuberculosis (TB) burden amongst the Saharia tribe of central India, there is hardly any study to investigate the impact of DOTS implementation on the magnitude of tuberculosis disease and the changes over time. This article present the findings of TB prevalence surveys conducted amongst this indigenous population in two different time periods to know the change in the prevalence of TB.

**Methods:**

A cross sectional survey was conducted among Saharia population in Shivpuri district, Madhya Pradesh during February 2013 to May 2013 and resurvey during March 2015 to July 2015. All individuals (≥15 years) were examined for chest symptoms suggestive of TB. Sputum samples were collected from all presumptive TB cases and were confirmed by laboratory examination by Ziehl-Neelsen smear microscopy and solid media culture methods. All detected cases were referred to health facility for anti-tuberculosis treatment as per RNTCP guidelines.

**Results:**

There was significant reduction (trend Chi square 19.97; OR = 1.521; *p* = 0.000) in the prevalence of TB at the endline (1995 per 100,000) as compared to baseline (3003 per 100,000). The reduction was significant among males as compared to females (OR 1.55; *p* = 0.000) and in the age group of 25–34 years (OR 2.0; *p* = 0.007) and 45–54 years (OR 4.39; *p* = 0.003). There was significant reduction in the prevalence in both smear (OR 1.29; *p* = 0.02) and culture positive (OR 1.57; *p* = 0.000) TB at the endline survey.

**Conclusion:**

The study findings highlight a reduction in the prevalence of TB among Saharia tribal population. Further studies are needed to identify the factors associated with reduction in prevalence among this population and also further surveys to monitor the prevalence trend over a period.

## Background

The tribal populations in India are one of the marginalised sections who are constrained by structural and cultural barriers in accessing health services. The magnitude of tuberculosis disease among vulnerable population and the changes over time are useful indicators for understanding the extent of TB transmission and for monitoring the effectiveness of TB control in the area. Information on these aspects is not available in tribal areas as it is difficult to obtain such information at the community level especially in a large country like India with limited resources. In India, the tribal population is 104 million constituting 8.6% of the total population and more than 70% of these reside in central Indian states of the country [1, 2]. Madhya Pradesh is one of the central Indian states accounting for 21.1% of tribal population with 46 ethnic groups. Of these, three are notified as Particularly Vulnerable Tribal Groups (PVTGs) [3]. The Saharia tribe is one among these PVTGs living in geographically isolated locations, working mainly as agriculture labours with very low socio economic living conditions. A very high prevalence of infection (20.4%) and TB disease (1518 per 100,000) has been reported among them [4, 5]. A recently conducted TB disease prevalence survey in Gwalior district show alarmingly high TB prevalence of 3294 per 100,000 in this community [6]. The high prevalence of PTB was found to be associated with malnutrition, poor housing conditions, alcoholism, tobacco smoking and history of asthma. HIV was not found to be a risk factor among them [7, 8].

Following a review of the National Tuberculosis Programme (NTP) in 1992, the Government of India initiated the Revised National Tuberculosis Control Programme (RNTCP) in 1993 and adopted internationally recommended Directly Observed Treatment Short-course (DOTS) strategy [9]. RNTCP after being successfully implemented for more than two decades had resulted in significant reduction of TB prevalence and it was demonstrated in Tamil Nadu [10]. With this background, we conducted periodic prevalence surveys to investigate the impact of DOTS implementation in this tribal community residing in hard to reach tribal areas of Shivpuri district of the state. This paper present the findings of the baseline and endline TB prevalence surveys conducted amongst them by the ICMR - National Institute for Research in Tribal Health (NIRTH), Jabalpur.

## Methods

### Study area and population

Saharia is the main tribal community in Shivpuri district, northwest Madhya Pradesh in central India. They generally inhabit small clusters of houses - a hamlet called as ‘Saharana’ outside the main village. A cross sectional sample survey was conducted among Saharia population in tribal dominated Pohri Block of the district during February 2013 to May 2013 and resurvey during March 2015 to July 2015 using the same methodology. The Directly Observed Treatment, Short-course (DOTS) has been implemented under the Revised National Tuberculosis Control Programme (RNTCP) in the study area since 2004. In addition to tuberculosis case detection under RNTCP at health facilities, a community survey for active tuberculosis detection was undertaken in the study area as epidemiological investigation.

### Sample size calculation

Assuming 25 % precision with 95% confidence level, design effect of two to adjust for clustering effect and 90 % sample coverage, the sample size of 9225 was calculated based on the reported prevalence of 1518/100,000 bacteriologically positive TB cases among tribal population [5]. Considering the average size of a Saharia hamlet as 300, it was estimated that about 45 villages would be required to be visited to cover the estimated sample of 9225 adult population (15 years and above).

### Sampling design and procedure

Villages were covered as the primary sampling units. Of the total 254 villages in Pohri Block, 128 were Saharia dominated (> 70% Saharia population) villages and 45 villages were randomly selected from these villages. Additional eight adjoining Saharia villages were also selected during the baseline survey to cover the sample of 9225 individuals. All these 53 villages were covered in the baseline and endline surveys.

### Data collection

Systematic communications with the local tribal leaders were established by the study team to inform them about the purpose of our survey. Study team also conducted community meetings prior to the survey. After this sensitization, house to house census was carried out by our trained field investigators and household information was recorded in an individual card in a pre-coded form. A baseline survey was conducted among all individuals aged 15 years and above to screen for symptoms suggestive of TB viz. cough for ≥2 weeks, fever ≥1 month, chest pain for ≥1 month and history of haemoptysis. Individuals remaining absent for symptom inquiry were revisited the same day or on subsequent days until at least 90% coverage was achieved. Persons having any of these symptoms were considered as chest symptomatic and two sputum specimens were collected for smear and culture examination. One sputum sample was collected on the spot and the overnight sample was collected in the morning in sterilized McCartney’s bottles from chest symptomatics as well as from asymptomatic individuals having a prior history of receiving anti TB treatment. The samples were sent to the ICMR-NIRTH laboratory, Jabalpur for smear and culture. ZN method was used to prepare smears from the concentrated samples. For culture examination, the sputum samples were kept in refrigerated condition till bacteriological investigations were carried out. The samples were processed and inoculated on 2 slopes of Lowensten Jensen (LJ) medium. The cultures were incubated and examined every week for the presence of mycobacterial colonies for a period of 8 weeks. In case of no growth or contamination, the cultures were discarded. *M. Tuberculosis* growth was confirmed using niacin test, 68 °C catalase test and growth on LJ with PNB (500 μg/ml). Individuals having positive smear and/or culture were considered to have tuberculosis and were referred to the health facility for anti-tuberculosis treatment as per RNTCP guidelines.

### Data management

All the completed cards and laboratory reports were scrutinized, checked for completeness and any incomplete forms sent back to the field for completeness. All the data were computerized in entry package developed on Census and Survey Processing System (CSPro) and SPSS was used for analysis. A pulmonary TB case was defined as an individual whose sputum was positive for AFB by ZN microscopy and/or growth of *M. tuberculosis* by culture examination. Prevalence of TB was estimated per 100,000 population and univariate analysis age, sex wise stratification was done. Statistical tool used was univariate analysis. Chi square test of significance was used to test differences in distribution of study population. Trend Chi square was used to test the prevalence differences in different surveys. The *p* value of < 0.05 was considered as the level of statistical significance.

### Ethical issues

The study was approved by the Institutional Ethics Committee at ICMR - National Institute of Research in Tribal Health, Jabalpur. The trained field investigators approached eligible individuals and explained the procedures, risks and benefits of the study in the local language. Written informed consent was obtained from all individuals willing to participate. All the patients diagnosed during the survey were referred to RNTCP and free counselling was provided to complete full course of the treatment.

## Results

### Coverage and TB cases

**Fig. 1:**
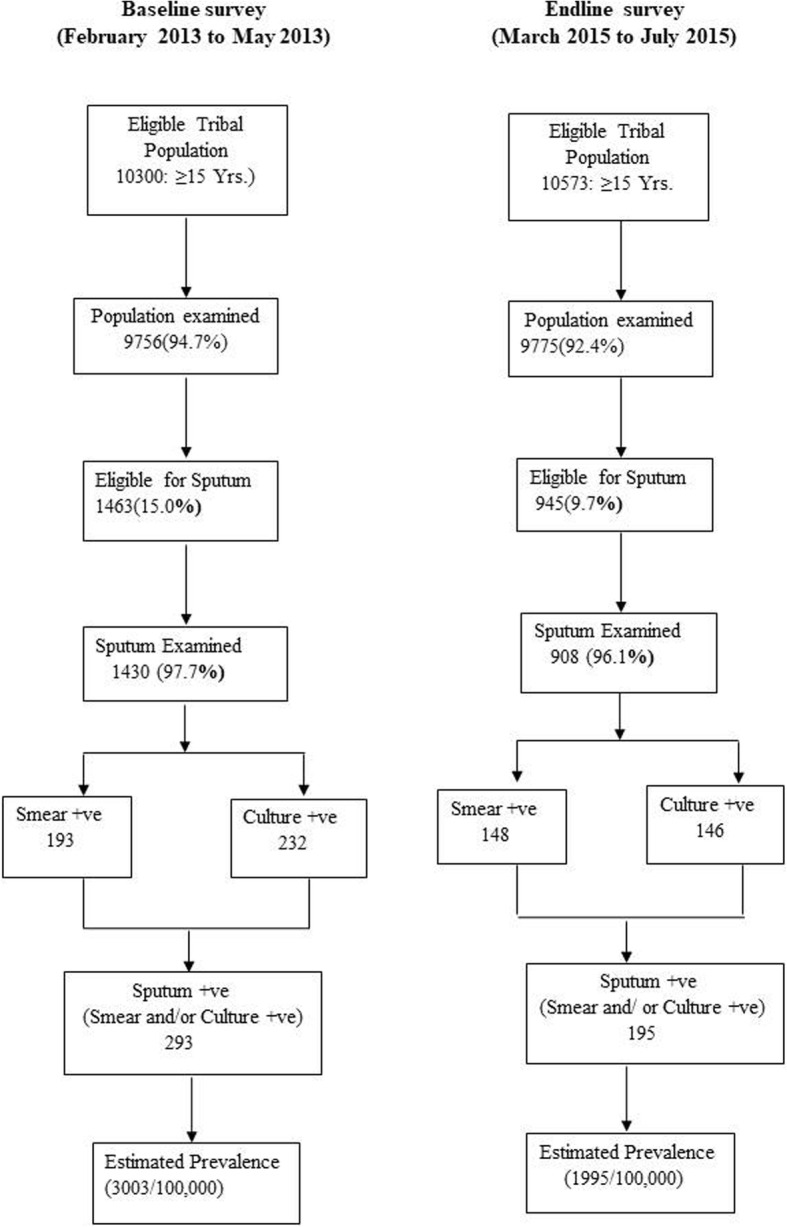
Process of baseline and endline prevalence surveys conducted among Saharia population, Shivpuri district, Madhya Pradesh

Of the total 10,300 and 10,573 individuals eligible for screening for baseline and endline surveys 9756 (94.7%) and 9775 (92.5%) individuals belonging to Saharia tribal community were screened (Table 1). Of the individuals screened for symptoms, 1463 (15.0%) and 945 (9.7%) were found to be symptomatic and sputum was collected from 1430 (97.7%) and 908 (96.1%) in baseline and end line surveys respectively. The coverage of > 90% was achieved during both the surveys. At the baseline and endline surveys, a total of 9756 and 9775 population was screened and 293 and 195 TB cases were detected respectively (Fig. 1), i.e. 50 Saharia individuals needed to be screened for detecting one PTB case in the endline survey as compared to 33 individuals in the baseline survey. The number of cases detected among male population was three times higher as compared to cases detected among females in both the surveys (231& 62 in baseline, 149& 46 in endline).

**Table 1:** Coverage of study population at baseline and endline surveys

	Baseline survey 2012–13			Endline survey 2014–15		
	No. eligible to examine	No. examined	Coverage (%)	No. eligible to examine	No. examined	Coverage (%)
Sex						
Male	5157	4781	92.7	5384	4700	87.3
Female	5143	4975	96.7	5189	5075	97.8
Age (years)						
15–24	2951	2853	96.7	2624	2531	96.5
25–34	2742	2616	95.4	3208	3015	94.0
35–44	1920	1783	92.9	1989	1837	92.4
45–54	1499	1391	92.8	1524	1367	89.7
≥55	1188	1113	93.7	1228	1025	83.5
Total	10,300	9756	94.7	10,573	9775	92.5

### Prevalence of pulmonary tuberculosis

**Fig. 2:**
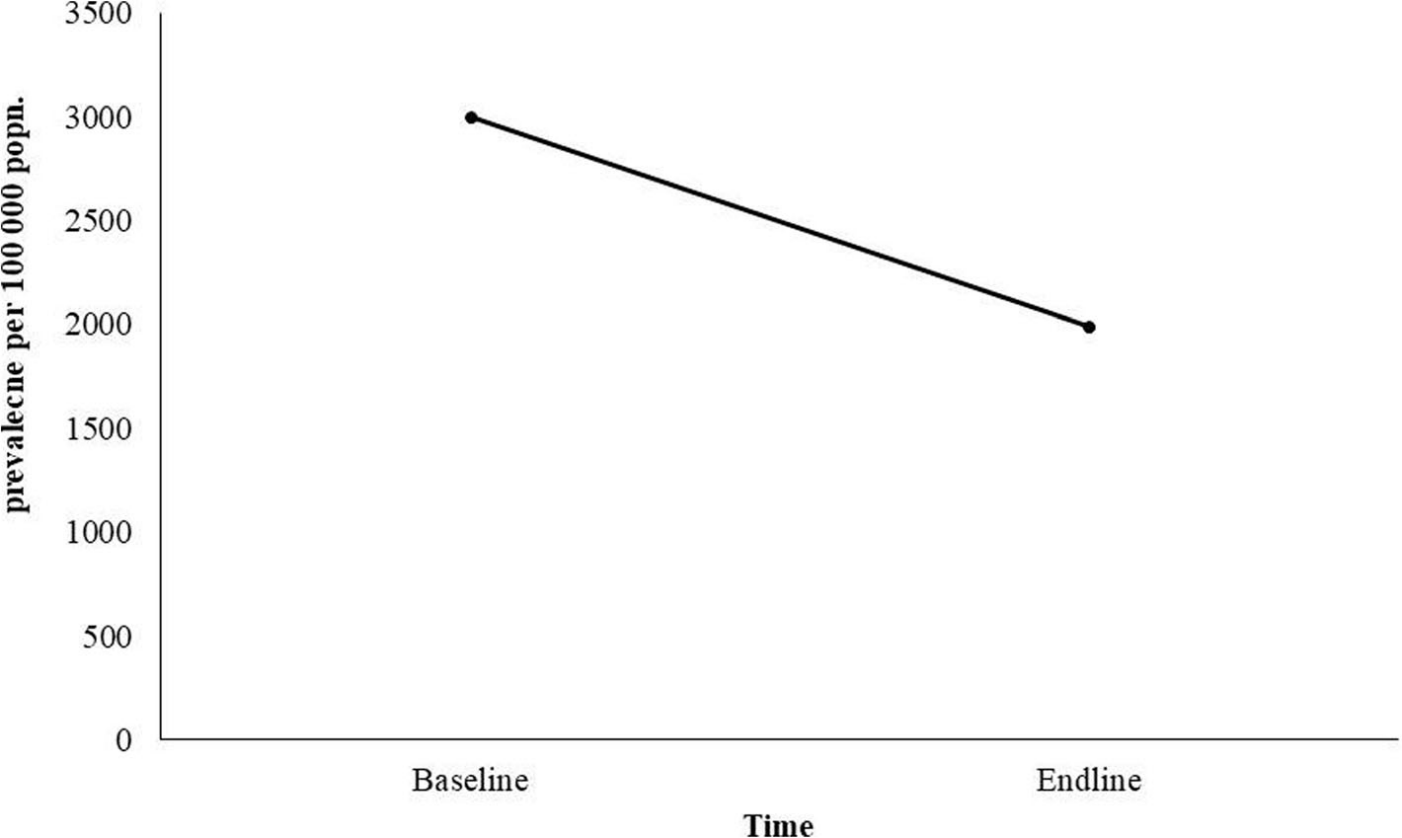
Prevalence of TB in two different surreys (base line and end line)

**Fig. 3:**
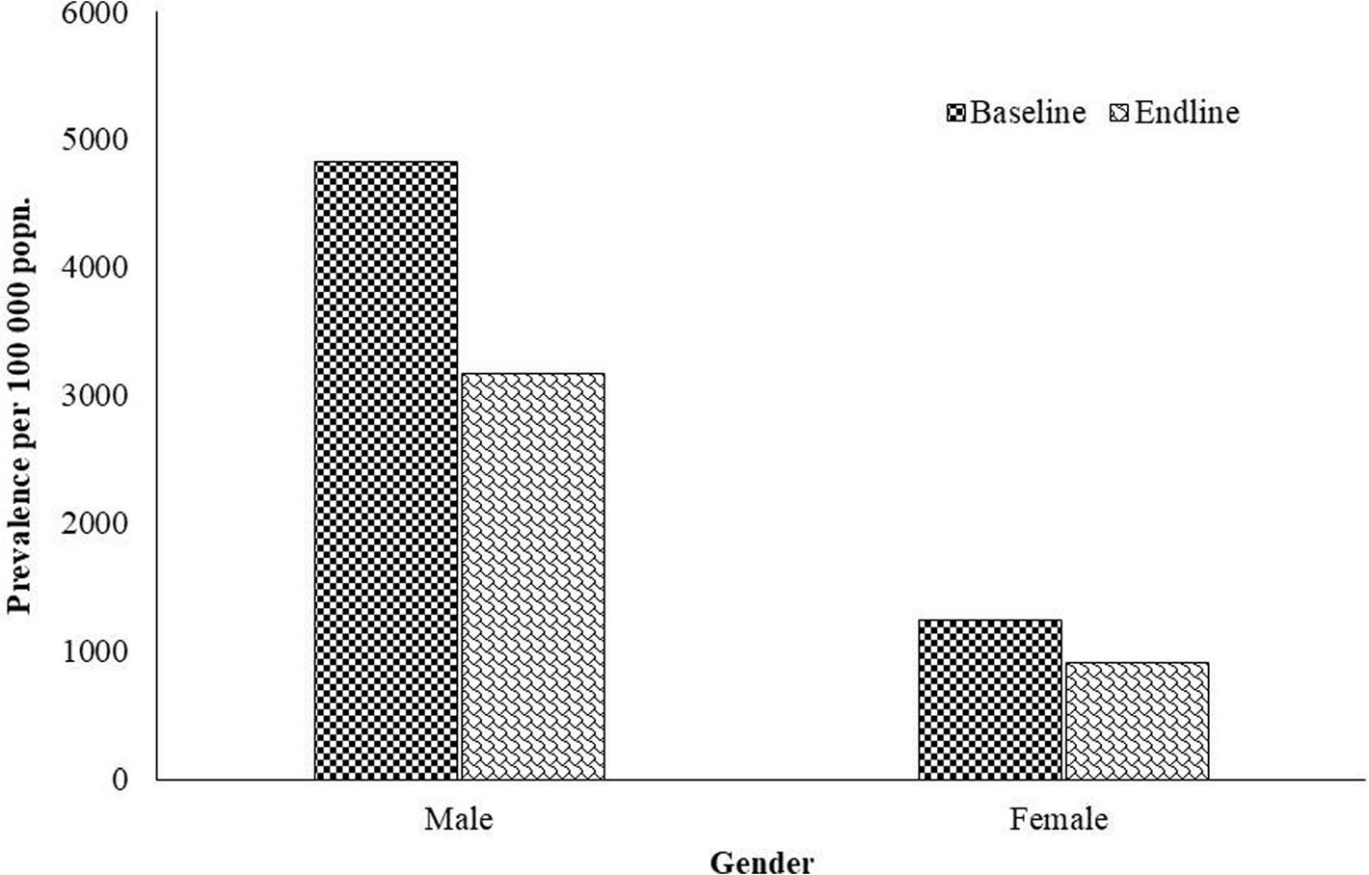
Prevalence of TB according gender in two different surveys

The overall prevalence among tribal population was found to be 3003 (95% Confidence Interval (CI): 2296–3710) per 100,000 in the baseline and 1995 (95% CI, 1323–2667) per 100,000 in the endline survey. There was significant reduction (trend Chi square 19.97; OR = 1.521; *p* = 0.000) in the prevalence of TB at the endline as compared to baseline (Fig. 2). In both the surveys, higher prevalence was observed among males compared to females (baseline 4832 vs 1246/ 100,000 population; endline 3170 vs 906/ 100,000 population). The reduction in prevalence was 1662 ((34.4%) among males and 340 (27.3%) among females (Fig. 3). There was significant prevalence reduction among males as compared to females (OR 1.55; *p* = 0.000). A significantly higher prevalence was consistently observed among males compared to females for smear, culture and bacteriologically positive PTB.

**Fig. 4:**
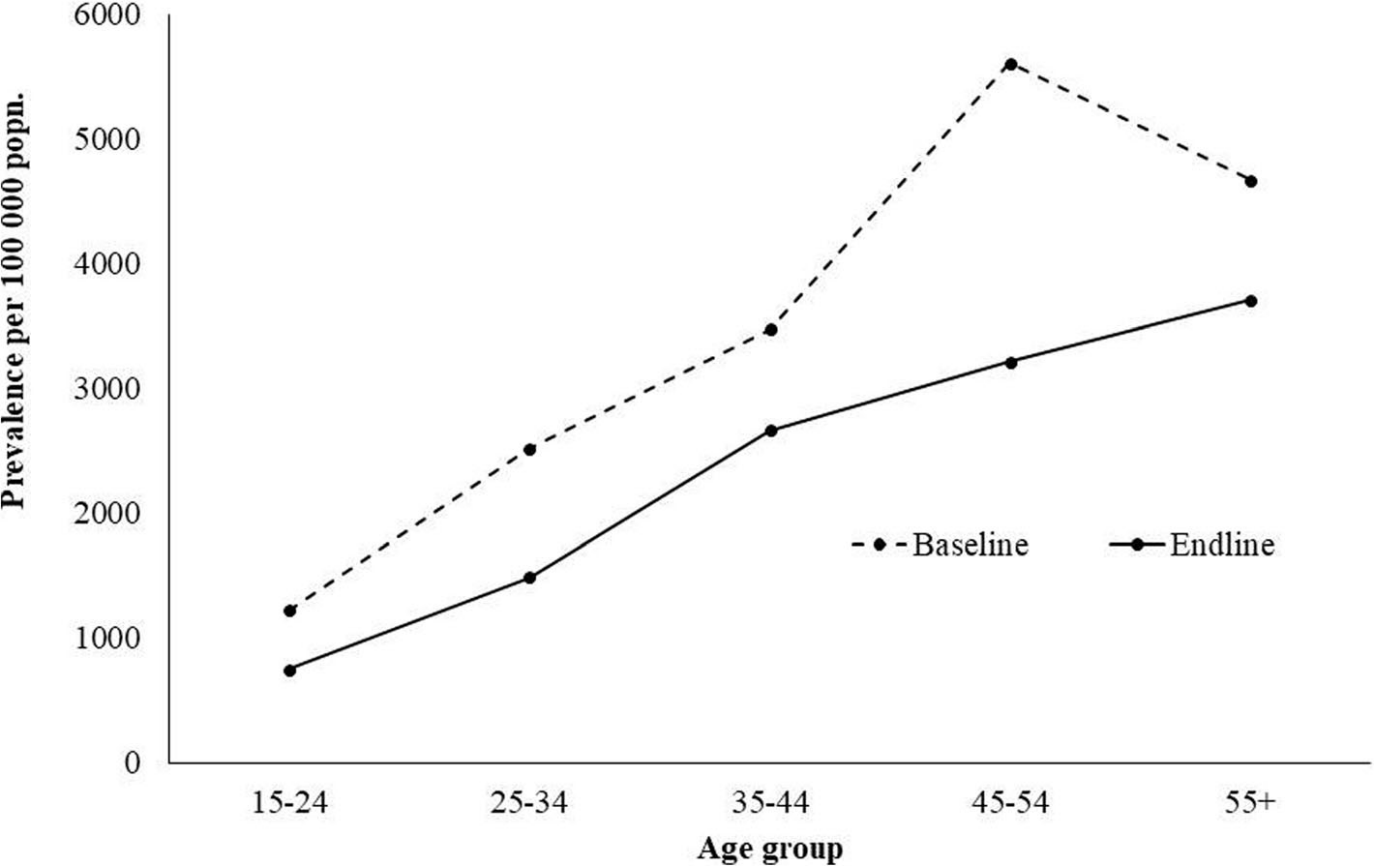
Prevalence of TB in two different surreys in different age group

The prevalence of smear positive PTB was estimated to be 1978 and 1514 per 100,000 during baseline and endline respectively. The prevalence of culture positive PTB was estimated to be 2378 and 1494 per 100,000 during baseline and endline respectively. There was significant reduction in the prevalence in both smear (OR 1.29; *p* = 0.02) and culture positive (OR 1.57; *p* = 0.000) TB at the endline as compared to baseline survey. The significant reduction in the prevalence in both smear and culture positive TB was also seen among males as compared to females (change in smear prevalence 25% vs 12.6%; culture positive 37.9% vs 31.2% respectively). The prevalence reduction was higher (more than 45%) among adult population aged 25–54 as compared to younger population. However, an increase in culture positive TB in the endline (2965 to 3024/100000) among population aged 55 years and above (Table 2). The prevalence of PTB increased with the age in both the baseline and endline surveys (Fig. 4). The reduction of TB was significant in the age group of 25–34 years (OR 2.0; *p* = 0.007) and 45–54 years (OR 4.39; *p* = 0.003).

**Table 2:** Prevalence of smear positive and culture positive TB at two different time point**s**

	Number examined		Number of Pulmonary TB		Prevalence Per 100,000 popn.		Change in prevalence	
	Baseline	Endline	Baseline	Endline	Baseline	Endline	No	%
Smear-positive								
Sex								
Male	4781	4700	156	115	3263	2447	816	25.0
Female	4975	5075	37	33	744	650	93	12.6
Age (years)								
15–24	2853	2531	24	17	841	672	170	20.2
25–34	2616	3015	42	37	1606	1227	378	23.6
35–44	1783	1837	45	39	2524	2123	401	15.9
45–54	1391	1367	51	31	3666	2268	1399	38.1
≥55	1113	1025	31	24	2785	2341	444	15.9
Culture positive								
Sex								
Male	4781	4700	185	113	3869	2404	1465	37.9
Female	4975	5075	47	33	945	650	294	31.2
Age (years)								
15–24	2853	2531	24	17	841	672	170	20.2
25–34	2616	3015	52	31	1988	1028	960	48.3
35–44	1783	1837	62	34	3477	1851	1626	46.8
45–54	1391	1367	61	33	4385	2414	1971	45.0
≥55	1113	1025	33	31	2965	3024	−59	−2.0

## Discussion

The salient finding from this study is the decreasing trend in prevalence of PTB in Saharia tribal community of central Indian state of Madhya Pradesh. Analysis of data on burden of disease caused by TB and the effectiveness of programmatic efforts to reduce this burden are crucial for public health action [11]. Further, this analysis is essential for programme evaluation which helps guide decisions about TB control management activities and policy. It supports TB control programme managers to monitor trends in the number and distribution of TB cases across the region. This enables TB control activities to report on the country’s TB epidemic and progress in reaching TB control goals and objectives. It also helps TB control programme managers to develop targeted national strategies and budget plans.

The current finding from community based prevalence survey highlight alarmingly high TB disease burden among Saharia tribe as compared to the national average and different population groups within the country [5]. This finding corroborate with the global evidences of high burden of TB among indigenous population [12]. The incidence of TB among indigenous peoples in Latin America was 1000/100000 and it was around 20 times higher than the incidence in the general population [13]. Among Yanomami people found the incidence of TB to be 2133/100000, or 37 times higher than in the surrounding non-indigenous population [14]. Indigenous people living in Cotopaxi Province in Ecuador’s highlands had TB prevalence rates reaching 6700/100000 [15].

It is encouraging that the repeat survey showed reduction in TB prevalence suggesting that efforts to improve TB control through active case finding was one of the important contributory factor in reducing the prevalence. The study from south India reported substantial reduction in TB prevalence in 5 years with DOTS implementation along with active case finding in the population [16, 17]. The tribal population in India also has access to free DOTS under RNTCP. This could be a plausible factor behind decreasing trend of TB among this vulnerable population. The analysis of national surveys conducted during 1990–2012 in Asia show 50% decrease in TB disease within a period of 10 years with some countries showing even greater decline with the available intervention techniques and methods [18].

However, the reduction among elderly population is less as compared to other age group particularly culture positive TB. It has been reported that smear positive TB is less in elderly and there are more chances for adverse drug reactions, default or death of newly diagnosed TB cases in this age group [19, 20]. Policy makers, in consultation with all the stakeholders, should consider developing specific intervention strategies for TB management in this population group. At the same time, the studies are also required to understand the effect of such interventions on the possible concentration of cases in this group. Given that TB prevalence was high among the elderly, there is a need to step up efforts to manage this vulnerable population.

The current study finding of reduction in TB prevalence occurred in a very short period, whereas the findings from four countries (Cambodia, China, the Philippines and Republic of Korea) demonstrated that approximately 50% reductions in TB prevalence can be achieved within 10 years [21–23]. Our findings could be understood in different programmatic context. India’s RNTCP’s DOTS strategy covered the entire country in 2006 and considering the high burden of TB among tribal population and structural barrier for diagnosis and treatment of TB in tribal areas, a tribal action plan was developed and implemented in parallel under RNTCP since 2005. Under the tribal action plan, strengthening and expansion of additional health facilities and involvement of community health workers was ensured. In addition, among Saharia population in Shivpuri district, IEC campaigns and door-to-door active TB case detection survey were implemented. The reduction in TB burden among this population could be due to the cumulative effect of all these interventions.

The study population is economically poor, socially marginalized, living far away from the main locality. There are numerous challenges that this population face in accessing healthcare facility which includes poverty, shortages of healthcare workers, non-availability of medicines, higher transportation cost, work absenteeism, lack of awareness on health facility and availability of treatment. Under the RNTCP Tribal Plan, additional decentralization of diagnosis and treatment services and special incentives to the health workers for tribal areas are provided [24]. The programme performance in the area has shown gradual improvement since implementation of RNTCP with an increase in the case detection rate from 121% in 2005 to 159% in 2017 and in the cure rate from 67% in 2005 to 83% in 2017 [25, 26]. Way back in 1989, Styblo had also highlighted the challenge before the policy makers that a significant cure rate among smear positive pulmonary TB cases is pre-requisite to achieve overall improvement in TB burden in developing countries [23]. It is important to have regular and effective monitoring in place in order to understand the effects of the intervention strategies – the improvements or otherwise in disease trend. The surveys carried out at regular intervals may also help in understanding the trend.

### Limitations of the study

The information on socioeconomic change in the community was not available and so its possible impact could not be assessed. Moreover, this evidence is generated from the Saharia tribe where HIV infection and MDR-TB are not highly prevalent [8, 27]. Our conclusion therefore, cannot be generalized to all areas, especially to those with a high prevalence of HIV infection or MDR-TB and non-tribes.

## Conclusion

In summary, our study has shown that there is a reduction in prevalence of TB among Saharia tribal population. The factors which led to the reduction in prevalence between the study periods need to be further studied. In general, there is a need for effective public health strategies for intervening disproportionate TB burden among Saharias, who live in resource poor settings.

## Abbreviations

AFB: Acid-Fast Bacilli
CSPro: Census and Survey Processing System
DOTS: Directly Observed Treatment Short-course
HIV: Human immunodeficiency virus
ICMR: Indian Council of Medical Research
IEC: Information, education and communication
LJ: Lowensten Jensen
MDR-TB: Multi-Drug-Resistant Tuberculosis
NIRTH: National Institute of Research in Tribal Health
NTP: National Tuberculosis Programme
PTB: Pulmonary tuberculosis
PVTG: Particularly Vulnerable Tribal Group
RNTCP: Revised National Tuberculosis Control Programme
SPSS: Statistical package for the social sciences
TB: Tuberculosis
ZN: Ziehl-Neelsen
